# Use of a Bronchial Blocker for One-Lung Ventilation in a Patient With Severe Airway Obstruction: A Case Report

**DOI:** 10.7759/cureus.98554

**Published:** 2025-12-05

**Authors:** Kaori Maeda, Eisuke Kako, MinHye So, Kazuya Sobue

**Affiliations:** 1 Department of Anesthesiology and Intensive Care Medicine, Nagoya City University Graduate School of Medical Sciences, Nagoya, JPN; 2 Department of Advanced Medical Nursing, Nagoya City University Graduate School of Nursing, Nagoya, JPN

**Keywords:** airway management, bronchial blocker, difficult airway management, one-lung ventilation, thoracic surgery, tracheal stenosis

## Abstract

One-lung ventilation (OLV) is often required during thoracic surgery to optimize surgical exposure and ventilation. However, in patients with severe airway obstruction, establishing OLV can be challenging because conventional lung isolation techniques, such as double-lumen tubes (DLTs), carry a high risk of airway injury. We report the case of a 73-year-old woman with a large goiter who underwent robot-assisted thoracoscopic thyroidectomy. Preoperative imaging revealed severe tracheal stenosis with a minimum diameter of 3.3 mm, precluding the use of a standard DLT. To achieve OLV with minimal airway manipulation, a single-lumen tube (SLT) with an internal diameter of 5.5 mm was used in combination with an intraluminal bronchial blocker (BB) inserted under bronchoscopic guidance. OLV was successfully maintained throughout the procedure without complications. The use of an intraluminal BB through a small-diameter SLT may represent a practical and less traumatic alternative for achieving lung isolation in patients with severe tracheal stenosis.

## Introduction

A difficult airway is defined as a clinical situation in which an experienced provider encounters difficulty with one or more of the following: face mask ventilation, direct or indirect laryngoscopy, tracheal intubation, supraglottic device placement, or surgical airway management [1]. Severe tracheal compression caused by an anterior cervical tumor can result in a difficult airway by obstructing airflow and compromising adequate oxygenation. When such a tumor extends into the thoracic cavity, one-lung ventilation (OLV) may be required to provide optimal surgical exposure. However, establishing OLV in these patients is particularly challenging because no standardized method for lung isolation exists. Standard double-lumen tubes (DLTs) or bronchial blockers (BBs) may not pass through the stenotic trachea, and the use of small-diameter single-lumen tubes (SLTs) carries a risk of mucosal injury in the fragile, narrowed airway. Furthermore, manipulation of an extraluminal BB or repeated repositioning of an SLT can exacerbate airway trauma [2]. Therefore, a technique that minimizes manipulation within the stenotic segment is desirable. The intraluminal placement of a BB through a small-diameter SLT may be a safer, less invasive approach to achieve OLV in such cases. Here, we report the successful use of this technique in a patient with severe tracheal stenosis undergoing robot-assisted thoracoscopic thyroidectomy.

## Case presentation

**Figure 1:**
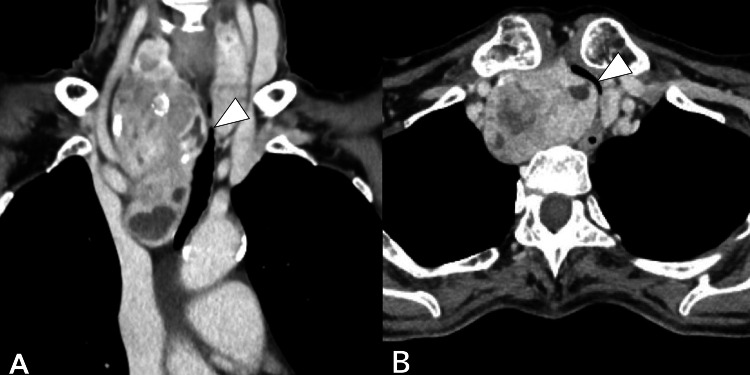
Preoperative CT Images Preoperative chest CT images with coronal view (A) and axial view (B) showing a massive right goiter descending from the right lobe of the thyroid gland to the middle mediastinum. Arrowheads indicate the narrowest parts of the trachea. CT: computed tomography

**Figure 2:**
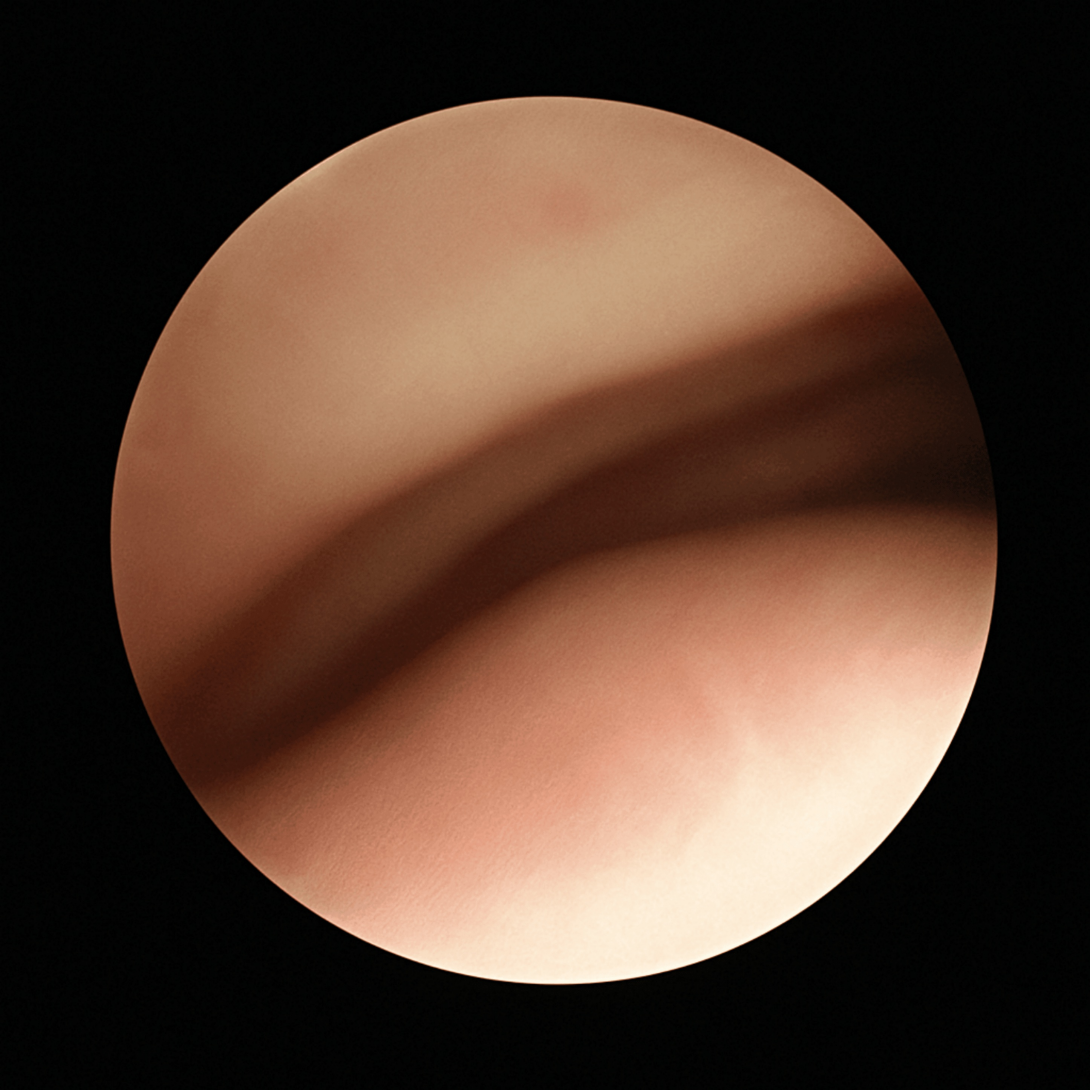
Preoperative bronchoscopy showing the narrow part of the trachea

A 73-year-old woman (height: 151 cm, weight: 36 kg) with a massive right-sided goiter extending from the right thyroid lobe to the middle mediastinum was scheduled for robot-assisted thoracoscopic thyroid tumor resection combined with a cervical procedure. Her medical history included ulcerative colitis treated with prednisolone. Computed tomography (CT) revealed a 103 × 54 × 41 mm goiter compressing the trachea from the right side, with the narrowest internal tracheal diameter measuring only 3.3 mm (Figure 1). Preoperative bronchoscopy demonstrated severe tracheal narrowing (Figure 2), though compression was reduced near the tracheal bifurcation. Despite the marked stenosis, the patient was asymptomatic during daily activities but preferred sleeping in the left lateral recumbent position.

Because airway management was expected to be difficult, general anesthesia was planned with venovenous extracorporeal membrane oxygenation (VV-ECMO) on standby. OLV was required for thoracoscopic resection. A DLT was unsuitable because its outer diameter exceeded that of the stenotic trachea. Therefore, OLV was planned using a BB inserted intraluminally through a small-diameter endotracheal tube. Although the goiter was mobile, placement of an endotracheal tube in the already compressed airway risked tracheal edema. To minimize this risk, we preoperatively identified the smallest endotracheal tube internal diameter (ID) that would permit the co-manipulation of a 1.8-mm fiberoptic bronchoscope (FOB) (LFP; OLYMPUS, Tokyo) and a BB (COOPDECH; 3.0-mm outer diameter, DAIKEN MEDICAL, Osaka). Hence, we used a microcuff endotracheal tube with a 5.5-mm ID (Avanos Medical Japan, Yokohama).

Upon arrival in the operating room, 4-Fr sheaths were inserted into both femoral veins for possible VV-ECMO initiation in case of airway collapse. After preoxygenation with 6 L/min oxygen, general anesthesia was induced using fentanyl (50 µg), remifentanil (0.2 µg/kg/min), and remimazolam (4 mg/kg/h). We combined fentanyl and remifentanil to stabilize circulation during the induction of anesthesia. Mask ventilation was easily achieved, after which rocuronium (30 mg) was administered. Intubation was performed smoothly using a 5.5-mm ID microcuff endotracheal tube and a McGrath video laryngoscope (blade size 3; Medtronic, Minneapolis). The goiter was mobile, and endotracheal tube insertion was considered feasible. A non-reinforced tube was selected to minimize the outer diameter while maintaining a sufficient ID for instrument passage. A 3.1-mm FOB (MAF-DM2; OLYMPUS, Tokyo) was used to guide the placement of the tube tip distal to the stenotic segment (Video 1). The BB was then advanced into the right main bronchus under bronchoscopic guidance using a 1.8-mm FOB, ensuring adequate ventilation of the left lung.

**Video 1:** Endotracheal tube positioning under bronchoscopic guidance

OLV was initiated with FiO₂ 1.0, peak inspiratory pressure (PIP) 35 cmH₂O, positive end-expiratory pressure (PEEP) 7 cmH₂O, and respiratory rate 12/min in the left lateral position. Although lung compliance was normal, a higher PEEP was applied to prevent atelectasis and maintain oxygenation, as effective recruitment maneuvers would be difficult through the narrow, high-resistance airway. The initial tidal volume was approximately 200 mL, with arterial blood gas values of pH 7.19, pCO₂ 69 mmHg, and pO₂ 422 mmHg. As the surgery progressed, a gradual increase in tidal volume and resolution of hypercapnia were observed (pH 7.30, pCO₂ 54.3 mmHg, pO₂ 82.1 mmHg), suggesting improved compliance of the dependent lung (Table 1).

**Table 1:** Intraoperative trends in ventilator settings and arterial blood gas analysis OLV: one-lung ventilation, FiO₂: fraction of inspired oxygen, PIP: peak inspiratory pressure, PEEP: positive end-expiratory pressure, RR: respiratory rate, TV: tidal volume, PO₂: partial pressure of oxygen, PCO₂: partial pressure of carbon dioxide

Time point	FiO₂	PIP (cmH2O)	PEEP (cmH2O)	RR (/min)	TV (mL)	pH	PO₂ (mmHg)	PCO₂ (mmHg)
Start of OLV	1.0	35	7	12	212	7.19	422	69
1 hour after OLV	1.0	35	7	12	281	7.26	86.2	59
2 hours after OLV	1.0	33	7	12	266	7.3	82.1	54.3

Following completion of the thoracic portion of the procedure, the BB was removed, and the cervical portion was performed in the supine position. After excision of the goiter, tracheal stenosis and compression were relieved. The patient was extubated immediately after surgery without airway obstruction, and her respiratory status remained stable. Postoperative recovery was uneventful, and the final pathological diagnosis was adenomatous goiter.

## Discussion

A mediastinal goiter is a high-risk factor for severe airway obstruction and a consequent difficult airway. Anesthesia management for patients with mediastinal tumor syndrome, a condition where a mediastinal tumor causes respiratory and circulatory dysfunction, is particularly challenging [3]. Thorough preoperative examination, evaluation, and preparation are required. When imaging reveals >50% airway compression or the patient has symptoms from airway narrowing, cardiopulmonary bypass is often recommended during anesthetic induction [3]. Similarly, Kim and colleagues recommended elective ECMO for patients with tracheal stenosis in which the stenosed segment was less than 5 mm in diameter, as identified by bronchoscopy or chest CT [4]. Having ECMO on standby, as in our case, is a prudent measure, especially as manual cardiopulmonary resuscitation lasting >30 minutes before ECMO initiation is associated with poor outcomes [5].

As noted, standard OLV options are limited in patients with severe tracheal stenosis. DLTs are often too large to pass the stenosis. Advancing a small-diameter SLT into a main bronchus is an alternative. Still, these SLTs are often too short for bronchial intubation, and repeated repositioning to achieve or release lung isolation risks mucosal injury. While the extraluminal placement of a BB has been advocated, this approach requires the blocker to pass directly through the narrowed segment. This maneuver has been reported to cause mucosal trauma [2]. In contrast, our intraluminal technique uses the SLT as a protective conduit, shielding the vulnerable mucosa from the BB during placement. Other alternatives for very narrow airways include using a Fogarty catheter as a blocker [6].

In this case, we achieve OLV even with an endotracheal tube with a 5.5 mm ID and a BB. The outer diameter of this tube was 7.3 mm, which exceeded the diameter at the narrowest part of the trachea (3.3 mm). However, tracheal stenosis was caused by compression from a goiter, which was determined to be mobile. Consequently, intubation was performed without difficulty. Anticipating the challenge of manipulating both a BB and an FOB simultaneously within the narrow tube, we conducted a preoperative simulation and confirmed the procedure's feasibility. This proactive step in risk mitigation was crucial for procedural success. We also confirmed the branching pattern of the right upper lobe bronchus on preoperative CT scans, which determined that lung isolation with the BB was feasible. As a result, the BB was placed in an appropriate position without any complications.

A key concern was whether sufficient ventilation could be achieved for an adult, given the high resistance created by the BB’s presence within the narrow endotracheal tube. In this case, despite requiring a high driving pressure of approximately 30 cmH₂O, we achieved a tidal volume of over 200 mL. These findings provide valuable information for future clinical practice. Given patient safety concerns and the possibility of uncontrolled respiratory acidosis, having ECMO on standby was an appropriate and necessary precaution.

## Conclusions

We describe a case in which OLV was achieved using an intraluminal BB in a patient whose trachea was severely compressed by a massive goiter. There are several methods for achieving OLV. However, this approach minimized manipulation of the stenotic region. To our knowledge, this is one of the few reports demonstrating the successful use of an intraluminal bronchial blocker through a 5.5 mm SLT in a patient with critical tracheal stenosis. This case highlights the feasibility and effectiveness of OLV with an intraluminal BB in a patient with an obstructed airway.
